# Comprehensive Echocardiography Interpretation Using Video and Multiview Vision-Language AI

**DOI:** 10.1016/j.jacasi.2026.06.039

**Published:** 2026-08-27

**Authors:** Ryo Takizawa, Chiemi Yamazaki, Satoshi Kodera, Tempei Kabayama, Shun Kitamura, Risa Kishikawa, Ryo Matsuoka, Megumi Hirokawa, Junichi Ishida, Koki Nakanishi, Hiroyuki Morita, Norihiko Takeda

**Affiliations:** a Department of Cardiovascular Medicine, The University of Tokyo Hospital, Tokyo, Japan; b Department of Clinical Laboratory, The University of Tokyo Hospital, Tokyo, Japan; c International University of Health and Welfare, Tokyo, Japan

**Keywords:** contrastive language-image pre-training (CLIP), medical AI, video language models

## Abstract

**Background:**

Echocardiography is essential for assessing cardiac structure and function, yet accurate interpretation requires specialized expertise, creating challenges in emergency care and in regions with limited access to experienced echocardiographers. Although artificial intelligence–based interpretation is increasingly studied, most existing models rely on still images or single views and do not reflect the video-based, multiview integration used in clinical practice.

**Objectives:**

The authors aim to develop and evaluate a multiview video-language model that integrates cardiac motion and multiple standard echocardiographic views.

**Methods:**

We trained a vision-language model on 577,061 transthoracic echocardiography videos paired with Japanese clinical reports from 46,852 examinations acquired at a single tertiary center (2015-2023). For each examination, features from 5 standard views (parasternal long-axis and short-axis; apical 2-, 3-, and 4-chamber) were aggregated. Performance was assessed in a retrieval task selecting the correct report from 16,436 candidate reports in the test cohort based on video input; the primary metric was correct match retrieval probability within the top 10 candidates (ie, R@10).

**Results:**

The image-based baseline achieved an R@10 of 1.3%. Using video input increased R@10 to 4.2%. Integrating 5 views further improved R@10 to 6.0% (95% CI: 5.5%-6.4%). Gains were greatest for findings that depended on temporal dynamics and multiview assessment, including left ventricular wall motion abnormality and left ventricular dilation.

**Conclusions:**

A multiview video-language framework improved report retrieval compared with conventional image-based approaches. These findings support the utility of video-based, multiview representation learning for echocardiographic report retrieval.

Echocardiography is a widely used, noninvasive method for diagnosing various cardiac conditions, including myocardial infarction, valvular diseases, and congenital heart defects.^1^^,^^2^ However, accurate interpretation of echocardiographic videos requires specialized expertise, which can be both time consuming and costly, especially in emergency care settings or in regions with limited access to trained specialists.^3^^,^^4^ These real-world limitations have increased interest in AI-based approaches that can support or partially automate echocardiographic interpretation.

In recent years, vision-language models capable of jointly processing images and text have advanced rapidly, enabling the alignment of echocardiographic images with their corresponding clinical reports.^5^^,^^6^ These vision-language models are progressively improving their ability to integrate textual information with diverse cardiac imaging patterns, and several models now demonstrate the capacity to reflect diagnostic features typically emphasized by experienced clinicians. EchoCLIP is a CLIP (Contrastive Language-Image Pre-training) model trained on 1,032,975 echocardiographic images paired with clinical reports from 224,685 cases.^7^^,^^8^ By learning to align numerical feature representations (“embeddings”) that summarize the clinically relevant visual patterns in echocardiographic images with corresponding representations of clinical reports, EchoCLIP can assess disease presence and severity based on the inferred similarity between imaging findings and textual descriptions. This CLIP-based approach provides a generalizable solution for interpreting diverse cardiac conditions. Furthermore, the ability of vision encoders to transform raw echocardiographic images into informative feature representations is crucial for developing multimodal large language models capable of generating clinical reports and comprehensive diagnostic reasoning.

Despite substantial progress in vision-language modeling for echocardiography, including recent multiview and video-based approaches such as EchoPrime, important challenges remain in fully understanding how best to leverage the unique characteristics of echocardiographic data.^9^ In particular, the relative contributions of temporal information captured in videos and the integration of multiple anatomical views have not been systematically disentangled or quantitatively evaluated. Unlike static imaging methods such as chest x-rays, echocardiograms capture the heart's rhythmic motion, an essential aspect for diagnosing certain conditions (eg, valvular disease with abnormal blood flow). Another key feature of echocardiography is its variety of views. Because the heart is a 3-dimensional organ, positioning the ultrasound probe at different angles yields distinct cross-sections. While there are dozens of potential views, the commonly used ones are obtained from 2 primary approaches: parasternal views, such as the long-axis (LAX) and short-axis (SAX) views, and apical views, including the 2-, 3-, and 4-chamber (2CH, 3CH, 4CH) views. These windows visualize different anatomical planes and offer complementary diagnostic information, suggesting that integrating information from multiple views may further enhance interpretation performance. In this study, we aim to enhance the interpretation capability of a CLIP model by leveraging these 2 characteristics of echocardiography data.

## Methods

### Study population and dataset preparation

We collected 69,482 transthoracic echocardiographic examinations from 34,155 patients who underwent imaging at The University of Tokyo Hospital between 2015 and 2023. Each examination was associated with a single clinical report, whereas multiple echocardiographic video clips from different standard views could be acquired for a single examination.

All echocardiographic videos were preprocessed using an internally developed view-classification convolutional neural network, which automatically assigned each video to one of the 5 standard views—LAX, SAX, 2CH, 3CH, or 4CH. In an independent validation dataset, this classifier achieved an overall accuracy of 97.8%, with area under the receiver-operating characteristic curve (AUC) values ranging from 0.994 to 1.000 across view categories (Supplemental Table 1). SAX images were defined as those acquired at the papillary muscle level. To ensure reliable view assignment, we excluded videos with poor classification confidence or those assigned to nonstandard echocardiographic views. Specifically, only videos classified into one of the 5 standard views with a probability of ≥0.9 were retained for model development. These curated videos were then used to construct the training datasets for the 3 baseline models: MultiVideo, SingleVideo, and SingleImage. The MultiVideo model used all available videos across the 5 standard views (LAX, SAX, 2CH, 3CH, and 4CH) for each case, whereas the SingleVideo and SingleImage models used only 4CH-view videos.

To prevent information leakage, dataset splitting was performed at the patient level, such that all echocardiographic examinations from a given patient were assigned exclusively to either the training, validation, or test set. To further assess model generalizability, we performed a patient-level split cohort analysis in which the dataset was divided into training (70%) and testing (30%) cohorts, ensuring no overlap of patients between cohorts. When ≥1 views were unavailable for a given examination, the model used the available videos without imputation, and study-level embeddings were computed by averaging the embeddings of the available clips.

To minimize the possibility of shortcut learning from nonphysiologic cues, all echocardiographic videos were spatially cropped to retain only the central imaging field. This preprocessing step removed peripheral screen elements such as patient identifiers, acquisition dates, vendor user-interface elements, and measurement overlays. In routine echocardiography systems, quantitative measurements and annotations are typically displayed on still frames rather than continuously within cine loops. Because our model was trained exclusively on echocardiographic videos rather than still images, these measurement values and annotations were not included in the model inputs. Consequently, the video inputs used for model development did not contain textual metadata or measurement overlays.

This study was approved by the Institutional Review Board of The University of Tokyo (approval No. 2024312NI-(1)). The requirement for informed consent was waived given the retrospective nature of the study.

### Model overview

To incorporate temporal information, we replaced the original image encoder of the CLIP architecture with a video encoder.^10^, ^11^, ^12^ The model was trained using contrastive learning on paired echocardiographic videos and clinical reports.^8^ Model performance was evaluated using cross-modal retrieval tasks: video-to-text retrieval, in which a query video is used to rank candidate reports and the matching report is identified, and text-to-video retrieval, in which a query report is used to rank candidate videos and the corresponding video is identified. In both settings, retrieval is performed by computing similarity in the shared embedding space learned during training, and accuracy is assessed based on how highly the correct paired sample is ranked.

### Contrastive learning

To train the model to associate echocardiographic videos with their corresponding clinical reports, we adopted a contrastive learning framework, similar to the strategy used in EchoCLIP. To optimize the shared embedding space between echocardiographic videos and clinical reports, we employed a symmetric contrastive objective that consists of 2 complementary components: a video-to-report term and a report-to-video term. Contrastive learning encourages the model to bring the embeddings of matching video-report pairs closer together, while pushing nonmatching pairs farther apart. For each batch of paired samples, the video encoder generates video embeddings and the text encoder generates report embeddings. Two complementary loss components are applied:

(1) Video-to-report loss: Encourages each video embedding to be most similar to its corresponding report embedding.
(2) Report-to-video loss: Encourages each report embedding to be most similar to its corresponding video embedding.

The final training loss is the average of these 2 components. The explicit mathematical definitions of the 2 losses are provided in the Supplemental Material (Equation S1: video-to-report loss; Equation S2: report-to-video loss).

Training was performed at the clip level, where each echocardiographic video clip was paired with its corresponding clinical report, allowing multiple clips from the same examination to form independent positive pairs with the same report, consistent with the training strategy used in EchoCLIP.^7^ Because multiple clips can originate from the same examination, clips from the same study may appear within the same batch and be treated as negatives in the contrastive objective. To mitigate this potential issue, we used a batch size of 64 for the video-based models, which reduces the likelihood that multiple clips from the same study are included in a single batch. Study-level aggregation was not used during training; instead, aggregation across multiple clips belonging to the same examination was applied only at inference time, where the embeddings from multiple videos were averaged to obtain the final representation used for retrieval.

All models were trained using 4 NVIDIA H100 GPUs. A batch size of 64 was used for video-based models and 2,304 for the image-based model. The learning rate was set to 1 × 10^−5^, with linear warm-up during the first 2,000 optimization steps followed by cosine annealing. Training each model required approximately 1 to 2 days.

### Model architecture

The overview of the model architecture is shown in Figure 1. For the video encoder, we employed a video vision transformer (ViViT) or video masked autoencoder (VideoMAE), which efficiently transforms a sequence of fixed-length frames (16 or 32 frames used) into a 512-dimensional embedding.^10^^,^^12^ The text encoder used was the BERT (Bidirectional Encoder Representations from Transformers) model.^13^ Since the clinical reports are written in Japanese, we used BERT Japanese V3, which was pretrained on a Japanese corpus.^14^ Additionally, while CLIP models such as EchoCLIP typically use 77 tokens for the text encoder, clinical reports in echocardiography often describe each symptom and item in detail, necessitating longer text inputs. Therefore, we adopted 256 tokens for the text encoder.

**Figure 1:**
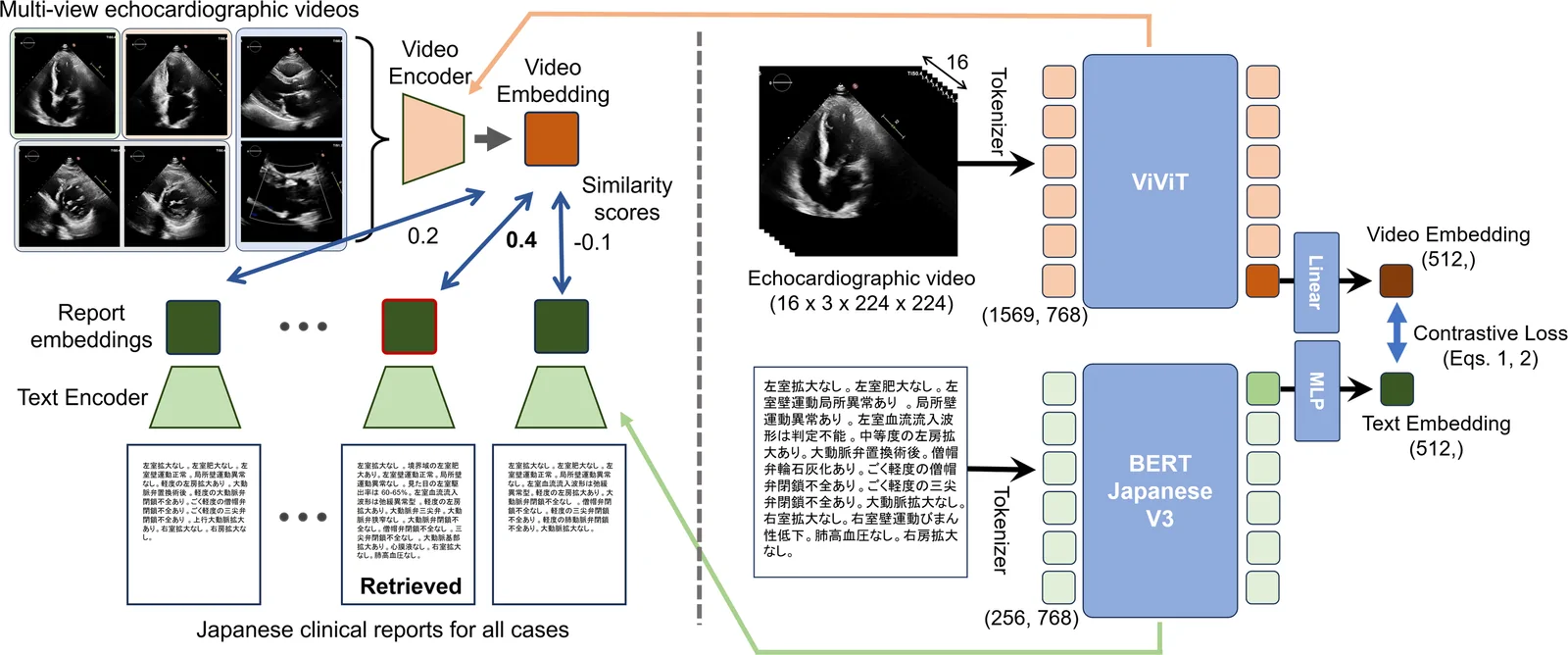
Multiview Echocardiography Interpretation Using a Video CLIP Model Multiview echocardiographic videos are processed using a video encoder, and corresponding clinical reports are encoded using a text encoder. The model is trained using contrastive learning to align video and report embeddings. During inference, similarity in the shared embedding space is used to retrieve the most appropriate clinical report for a given echocardiographic case, enabling automated interpretation based on multiview video input. CLIP = Contrastive Language-Image Pre-training; MLP = multilayer perceptron; ViViT = video vision transformer.

### Baseline models

We evaluated the retrieval performance of the proposed multiview video-input model (MultiVideo) by comparing it with 2 ablation models: a single-view video-input model (SingleVideo) and a single-view image-input model (SingleImage), the latter corresponding to EchoCLIP. Ablation models in this context refer to simplified comparison models designed to isolate the effect of removing either video information or multiview information.

SingleVideo shares the same architecture as MultiVideo and is trained exclusively on 4CH-view videos, which were selected because the 4CH view is the most widely available and has been commonly used in prior CLIP-based echocardiography studies such as EchoCLIP. In contrast, the MultiVideo-4CH model is trained using multiview video inputs but evaluated using only 4CH-view videos at test time, thereby enabling assessment of the effect of multiview training under a single-view (4CH-only) inference setting.

SingleImage replaces the video encoder with the ConvNeXt-Base image encoder.^15^ The training dataset for SingleImage only includes the 4CH view, and instead of randomly selecting a single frame from the cardiac cycle, a representative frame was sampled following previously published approaches to ensure consistency with prior work.

To ensure a fair comparison, all models used the same text encoder and were trained from scratch, meaning that no pretrained weights were used and all model parameters were initialized anew before training.

For the retrieval, both SingleVideo and SingleImage used only 4CH-view videos as input. Unlike the video-based models, SingleImage computed the mean of all image embeddings extracted from every frame across the multiple videos, following the approach used in the EchoCLIP study.

### Pretraining conditions

To examine how the choice of pretrained representations influences retrieval performance, we evaluated multiple pretraining strategies for both image-based and video-based encoders. For the image-based model (SingleImage), we applied pretrained weights derived from large-scale image datasets, including ImageNet-21k and LAION-2B.^16^^,^^17^ For the video-based model, we evaluated pretraining using Kinetics-400 and VideoMAE2, the latter of which differs from ViViT in architecture and processes 16 frames per video clip.^12^^,^^18^

All pretrained configurations were applied consistently across model comparisons, and no additional fine-tuning modifications were introduced other than those described in the earlier “Model Architecture” and “Baseline Models” sections, allowing isolation of the effect of pretraining.

### Model evaluation: multiview video report retrieval

The video encoder and text encoder, trained through contrastive learning, are used to retrieve the most appropriate report from a set of candidate reports based on the similarity of embeddings during interpretation. Each query examination was evaluated against 16,436 candidate reports in the test subset. These reports corresponded to examinations with available 4CH-view videos included in the comparative retrieval analyses. These examinations comprised 232,682 video clips across the 5 standard echocardiographic views.

For each finding or report descriptor, its existence and severity (eg, mild/moderate/severe) are associated with corresponding text, which is then converted into embeddings by the text encoder. For analyses involving the presence of clinical findings (eg, valvular diseases and chamber dilation), a finding was considered present if it was described as trivial or greater severity in the clinical report.

In echocardiography, multiple videos from different views are typically acquired for each case, and physicians generate a single report by integrating information across these views. In this study, all available echocardiographic videos for a given case are individually converted into embeddings, and their average is computed to obtain the overall video embedding. The similarity between this video embedding and the corresponding report embedding is then calculated and used to retrieve clinical reports. Conversely, it is also possible to retrieve the case with the most relevant echocardiographic videos for a given report. The entire report retrieval process based on multiview video interpretation is summarized in Supplemental Equation 3.

### External evaluation using the EchoNet-Dynamic dataset

To assess the generalizability of the proposed model beyond the internal test cohort, external evaluation was performed using 100 cases from the EchoNet-Dynamic dataset. Each case consisted of an apical 4CH echocardiographic video and corresponding quantitative measurements, including end-diastolic volume (EDV), end-systolic volume (ESV), and ejection fraction (EF). Reports were generated using the VideoMAE2-pretrained video model, SingleVideo model, and SingleImage model.

For left ventricular (LV) dilation assessment, reference categories were derived from EDV measurements as follows: normal (<110 mL), mild (110-139 mL), moderate (140-169 mL), and severe (≥170 mL). For LV wall motion assessment, reference categories were derived from EF measurements as follows: normal (≥55%), mildly reduced (50%-54%), and reduced (<50%). Report-derived classifications were compared with these reference categories using accuracy and weighted kappa (κ). The 95% confidence intervals were estimated using nonparametric bootstrap resampling with 2,000 iterations.

### Expert evaluation of generated reports

To assess the clinical quality of generated reports, 3 board-certified cardiologists independently reviewed reports generated by the VideoMAE2-pretrained video model from the same 100 EchoNet-Dynamic cases.

Reports were evaluated using 3 domains: diagnostic accuracy, completeness, and clinical usefulness. Diagnostic accuracy was selected because clinical accuracy has been used as a key endpoint in physician-based evaluations of AI-assisted report generation.^19^ Completeness was included because it is recognized as an essential component of report quality in studies of automated medical report generation.^20^ Clinical usefulness was assessed to evaluate the practical value of AI-generated reports as decision-support tools in echocardiographic interpretation, consistent with recent studies emphasizing the role of AI as an assistive technology in clinical workflows.^21^

Each domain was scored using a 5-point Likert scale (1 = poor, 2 = fair, 3 = acceptable, 4 = good, and 5 = excellent). Results were summarized as mean ± standard deviation and median (interquartile range).

### Statistical analysis

Continuous variables are presented as mean ± SD or median (IQR), as appropriate. Categorical variables are presented as counts and percentages.

Model performance was evaluated using mean cross-modal retrieval rank (MCMRR) and recall at rank *K* (R@1, R@5, and R@10) for both video-to-report (V→R) and report-to-video (R→V) retrieval tasks. MCMRR was used to assess overall retrieval ranking performance, whereas R@K was used to quantify the probability of retrieving the correct match within the top *K* candidates.

We estimated the 95% confidence intervals for retrieval metrics using nonparametric patient-level bootstrap resampling with 2,000 iterations. Pairwise comparisons between models were performed by calculating bootstrap-estimated differences with corresponding 95% CIs.

Finding-level diagnostic performance was assessed using sensitivity, specificity, positive predictive value, negative predictive value, and Cohen's κ for each predefined echocardiographic finding. Corresponding 95% CIs were estimated using the same patient-level bootstrap procedure.

The relationship between finding prevalence and retrieval performance (ie, R@10) across findings was assessed using Spearman rank correlation.

All retrieval analyses were performed on the held-out test cohort generated from the patient-level split cohort design. All statistical analyses were performed using Python version 3.10 and relevant scientific computing libraries.

## Results

### Dataset characteristics

The distribution of patients, examinations, echocardiographic videos, patient sex and age, and echocardiographic views across the training, validation, and test cohorts is summarized in Table 1. Each examination was associated with a single clinical report; therefore, the number of reports corresponded to the number of examinations in each cohort.

**Table 1:** Baseline Characteristics of the Study Cohorts

	Train (n = 23,057)	Valid (n = 854)	Test (n = 10,244)	Test Used (n = 8,809)^a^
No. of examinations	46,852	1,798	20,832	16,436
Age, y, median (IQR)	74.0 (57.0-84.0)	73.0 (57.0-83.8)	74.0 (58.0-84.0)	74.0 (58.0-83.0)
Male	12,136 (52.6%)	429 (50.2%)	5,323 (52.0%)	4,645 (52.7%)
Female	10,920 (47.4%)	425 (49.8%)	4,918 (48.0%)	4,162 (47.3%)
Total videos, n	577,061	22,060	256,057	232,682
LAX-view videos, n	155,418	6,049	68,902	58,139
SAX-view videos, n	147,661	5,707	65,754	58,569
2CH-view videos, n	50,571	1,945	22,296	20,871
3CH-view videos, n	81,119	2,971	35,883	31,881
4CH-view videos, n	142,292	5,388	63,222	63,222
LV dilation	5,510 (11.8%)	293 (16.3%)	2,361 (11.3%)	1,806 (11.0%)
LV wall motion abnormality	7,231 (15.4%)	348 (19.4%)	3,279 (15.7%)	2,517 (15.3%)
LV asynergy	4,234 (9.0%)	182 (10.1%)	1,960 (9.4%)	1,595 (9.7%)
Aortic stenosis	1,768 (3.8%)	65 (3.6%)	679 (3.3%)	583 (3.5%)
Aortic regurgitation	20,434 (43.6%)	778 (43.3%)	8,914 (42.8%)	6,976 (42.4%)
Mitral regurgitation	38,960 (83.2%)	1,505 (83.7%)	17,334 (83.2%)	13,839 (84.2%)
Tricuspid regurgitation	42,869 (91.5%)	1,675 (93.2%)	19,056 (91.5%)	15,117 (92.0%)
RV dilation	2,924 (6.2%)	107 (6.0%)	1,299 (6.2%)	1,024 (6.2%)

Values are n (%) unless otherwise indicated. Percentages for sex are calculated based on the number of patients in each cohort, whereas percentages for clinical findings are calculated based on the number of examinations in each cohort.

CH = chamber; LAX = long axis; LV = left ventricle; RV = right ventricle; SAX = short axis.

a The “test used” subset represents a subset of the test cohort consisting of examinations with available apical 4-chamber view videos included in the comparative retrieval analyses.

Among the 69,482 echocardiographic examinations included in this study, those that passed view-classification thresholds were allocated on a per-patient basis into training, validation, and test cohorts at proportions of 67.4%, 2.6%, and 30.0%, respectively, based on a patient-level split cohort design, corresponding to 46,852 examinations in the training cohort, 1,798 in the validation cohort, and 20,832 in the test cohort.

Age distribution was similar across cohorts, with a median age of 74.0 years in both the training and test cohorts. Overall, the sex distribution was balanced across cohorts. In the training cohort, 12,136 patients were male and 10,920 were female; in the test cohort, 5,323 patients were male and 4,918 were female. A small number of patients had unspecified or other sex recorded (1 patient in the training cohort and 3 patients in the test cohort).

For evaluation of model performance, we focused on examinations with an available 4CH view to enable fair comparison across models. Among the 20,832 examinations in the test cohort, 16,436 examinations contained a 4CH view and were included in the test subset used for model evaluation (denoted as “test used” in Table 1) for comparative retrieval analyses between multiview and single-view approaches.

Across the training dataset, the MultiVideo model was trained on a total of 577,061 videos aggregated from the 5 standard views (LAX, SAX, 2CH, 3CH, and 4CH), whereas the SingleVideo and SingleImage models were trained on 142,292 videos derived exclusively from the 4CH view.

The view-classification convolutional neural network used during preprocessing demonstrated high performance across all view categories (Supplemental Table 1), suggesting that view assignment errors were unlikely to substantially influence downstream retrieval performance.

### Model comparison

Table 2 shows the retrieval accuracy of the proposed model and the 2 ablation models. Accuracy was evaluated using MCMRR and recall at rank *K* (R@K), including R@1, R@5, and R@10. MCMRR represents the mean rank at which the correct report appears when all 16,436 reports are sorted by similarity, while recall at *K* (R@*K*) indicates the percentage of cases where the correct report is ranked within the top *K* positions.

**Table 2:** Model Performance in Patient-Level Split Cohort (70%/30%)

Model (Train, Test)	MCMRR ↓		R@1 (%) ↑		R@5 (%) ↑		R@10 (%) ↑	
	V→R	R→V	V→R	R→V	V→R	R→V	V→R	R→V
MultiVideo (multiview train, multiview test)	1,584 (1,543-1,625)	1,552 (1,511-1,593)	0.8 (0.7-1.0)	1.0 (0.8-1.1)	3.5 (3.2-3.8)	3.6 (3.3-4.0)	6.0 (5.5-6.4)	6.0 (5.6-6.4)
MultiVideo-4CH (multiview train, 4CH-only test)	1,887 (1,843-1,935)	1,851 (1,808-1,898)	0.7 (0.5-0.8)	0.6 (0.5-0.7)	2.7 (2.5-3.0)	2.2 (2.0-2.4)	4.6 (4.3-4.9)	4.0 (3.7-4.4)
SingleVideo (4CH-only train, 4CH-only test)	1,918 (1,877-1,966)	1,977 (1,934-2,026)	0.7 (0.6-0.8)	0.5 (0.4-0.6)	2.5 (2.3-2.8)	1.8 (1.6-2.0)	4.2 (3.9-4.5)	3.2 (2.9-3.5)
SingleImage (4CH-only train, 4CH-only test)	3,768 (3,700-3,840)	3,488 (3,424-3,557)	0.2 (0.1-0.2)	0.2 (0.1-0.2)	0.7 (0.6-0.9)	0.9 (0.7-1.0)	1.3 (1.2-1.5)	1.6 (1.4-1.8)

Model performance in the patient-level split cohort (70% training, 30% testing) with no overlap of patients between datasets. The proposed multiview video model (MultiVideo) was compared with baseline models (MultiVideo-4CH, SingleVideo, and SingleImage). Performance is reported for video-to-report (V→R) and report-to-video (R→V) retrieval tasks. Mean cross-modal retrieval rank (MCMRR) indicates the average rank of the correct match (lower is better), and recall at rank *K* (R@K) indicates the proportion of correct matches within the top *K* results (higher is better). Values are presented as point estimates with 95% confidence intervals, estimated using nonparametric patient-level bootstrap resampling with 2,000 iterations. Pairwise bootstrap comparisons between models are provided in Supplemental Table 2.

As shown in Table 2, the model with the highest retrieval performance was the multiview video model (MultiVideo), achieving an R@10 of 6.0% (95% CI: 5.5%-6.4%) and an MCMRR of 1,584 (95% CI: 1,543-1,625) for video-to-report retrieval. The next highest was the MultiVideo-4CH model, followed by the 4CH-view-only video model (SingleVideo), and the 4CH-view-only image model (SingleImage). Across all evaluation metrics (MCMRR, R@1, R@5, and R@10), the MultiVideo model consistently achieved the best performance. Pairwise bootstrap comparisons demonstrated consistently favorable differences for MultiVideo relative to the comparison models across most retrieval metrics (Supplemental Table 2). The most significant improvement in retrieval accuracy was observed when switching from image-based to video-based input, with R@10 increasing from 1.3% to 4.2% and a corresponding reduction in MCMRR. Incorporating multiple views improved performance further, with R@10 increasing to 6.0% and MCMRR decreasing accordingly.

To further evaluate the contribution of multiview information, we also compared the performance of MultiVideo when restricted to the 4CH view at inference (MultiVideo-4CH) with that of SingleVideo (Figure 2). Their similar results suggested minimal knowledge transfer from multiview training, indicating that the primary benefit of multiview lies in providing diverse information during inference.

**Figure 2:**
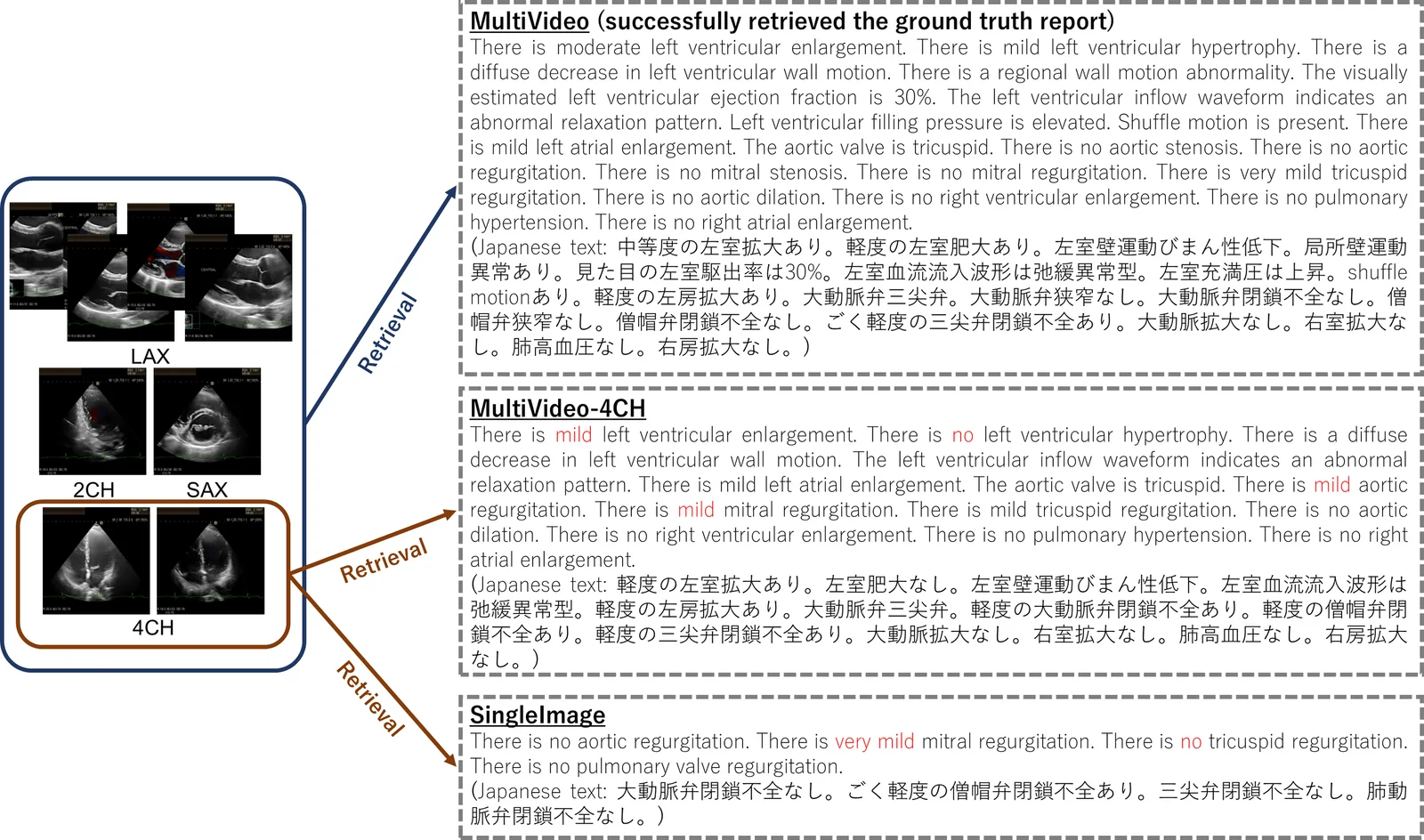
Comparison of Retrieved Clinical Reports Across Model Variants An example of the most similar clinical reports retrieved from 16,436 candidates for a specific echocardiographic case by SingleImage, MultiVideo-4CH, and MultiVideo. Reports are selected based on similarity to the input video. The multiview video model shows improved agreement with the ground-truth report compared with single-view video and image-based models. Text in red denotes discrepancies from the ground truth. 2CH = 2-chamber view; 4CH = 4-chamber view; LAX = long-axis view; SAX = short-axis view.

We additionally examined the distribution of ranks for the correct report across models (Figure 3). The multiview video model demonstrated a clear shift toward lower ranks compared with all baseline models, indicating more frequent retrieval of correct reports at higher positions.

**Figure 3:**
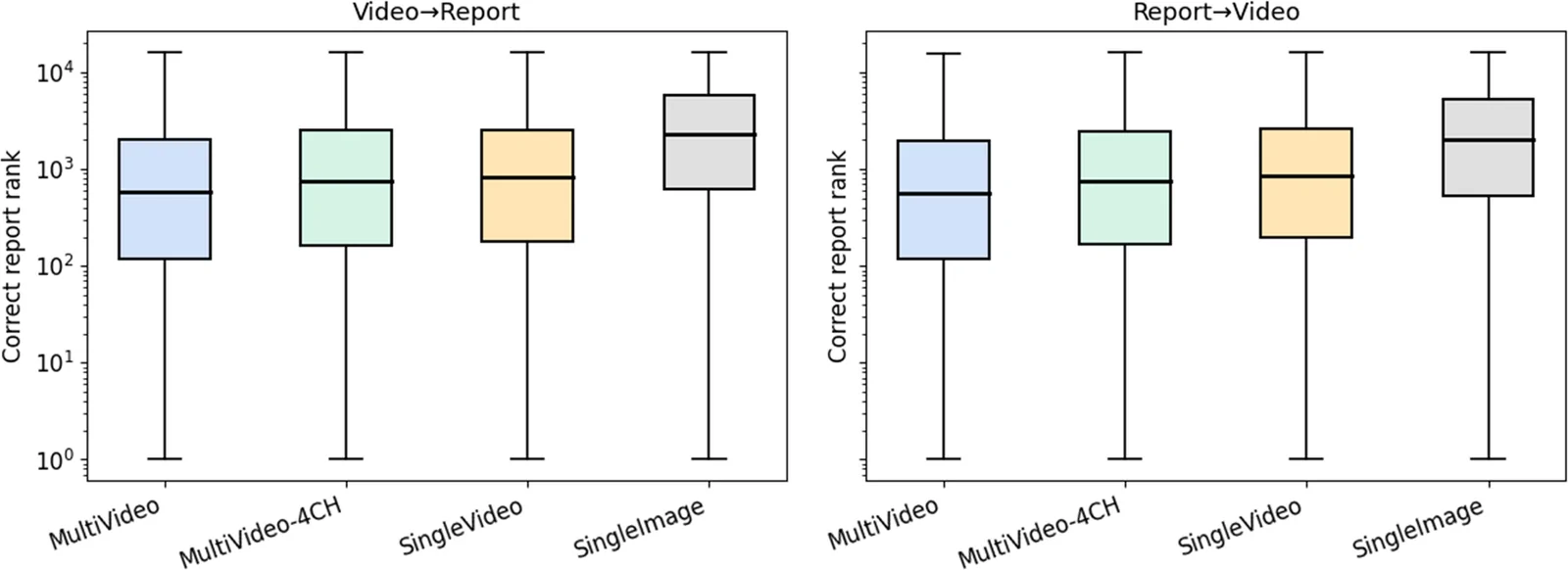
Rank Distribution of Correct Report Retrieval Distribution of ranks for the correct report across models, shown separately for video-to-report and report-to-video retrieval tasks. Results are shown as median (center bar), interquartile range (box), and full range (error bars). Lower ranks indicate better retrieval performance. The multiview video model demonstrates a shift toward lower ranks compared with baseline models, indicating more accurate retrieval of correct reports. 4CH = 4-chamber view.

We further evaluated model performance as a function of the number of available views (Supplemental Figure 1). Retrieval performance (R@10) generally increased as the number of views increased from 1 to 5. However, variability was observed within each group, with overlapping performance distributions across different view combinations.

Retrieval performance was further analyzed according to the number and combination of available echocardiographic views for selected clinical findings (Supplemental Figure 2). For each finding, R@10 values were calculated across all view combinations and summarized by the number of available views. Across findings, retrieval performance increased with the number of available views, with variability observed across different view combinations at each view count.

### Retrieval performance stratified by clinical findings

We additionally evaluated finding-level agreement between the ground-truth and retrieved reports for selected clinically relevant findings (Table 3), including LV dilation, wall motion abnormality, regional asynergy, major valvular diseases, and right ventricular (RV) dilation. Across most findings, the MultiVideo model achieved the highest agreement rates, followed by MultiVideo-4CH, SingleVideo, and SingleImage. The performance advantage of MultiVideo was most apparent for LV dilation, wall motion abnormality, and RV dilation. Agreement rates were generally similar across models for certain findings, such as tricuspid regurgitation.

**Table 3:** Finding-Level Agreement of Analysis

Model (Train, Test)	LV Dilation (n = 1,806)	LV Wall Motion (n = 2,517)	Asynergy (n = 1,595)	AS (n = 583)	AR (n = 6,976)	MR (n = 13,839)	TR (n = 15,117)	RV Dilation (n = 1,024)
MultiVideo (multiview train, multivideo test)	21.4 (19.2-23.4)	35.8 (33.5-38.2)	25.5 (23.2-27.7)	6.3 (4.4-8.5)	26.2 (25.1-27.3)	53.8 (52.8-54.7)	53.4 (52.2-54.3)	38.2 (34.2-42.4)
MultiVideo-4CH (multiview train, 4CH-only test)	20.5 (18.4-22,5)	33.3 (31.1-35.7)	24.2 (22.1-26.4)	6.0 (3.9-8.3)	24.9 (23.9-26.0)	52.3 (51.4-53.3)	53.3 (52.4-54.2)	36.0 (32.7-39.4)
SingleVideo (4CH-only train, 4CH-only test)	15.8 (14.0-17.6)	30.8 (28.5-33.2)	22.6 (20.3-24.8)	5.7 (3.9-7.7)	21.7 (20.7-22.7)	52.4 (51.5-53.4)	55.3 (54.4-56.2)	31.1 (27.5-34.7)
SingleImage (4CH-only train, 4CH-only test)	14.2 (12.6-15.9)	22.8 (21.1-24.6)	21.4 (19.4-23.3)	2.6 (1.2-4.0)	22.0 (20.9-23.0)	49.8 (48.9-50.7)	49.4 (48.5-50.3)	28.8 (25.6-31.8)

Values represent exact agreement rates (%) between the ground-truth report and the retrieved report among examinations positive for each finding. Positive findings were defined as trivial-or-greater abnormalities. Exact agreement was defined as matching ordinal severity labels between the ground-truth and retrieved reports. Values are presented as point estimates with 95% CIs in parentheses, estimated using patient-level bootstrap resampling with 2,000 iterations, n indicates the number of ground-truth positive examinations for each finding. LV wall motion indicates any wall motion abnormality, whereas asynergy refers to regional wall motion abnormalities.

AS = aortic stenosis; AR = aortic regurgitation; CH = chamber (view); LV = left ventricular; MR = mitral regurgitation; RV = right ventricular; TR = tricuspid regurgitation.

To further assess whether clinically relevant findings were preserved in the retrieved reports, we evaluated finding-level diagnostic performance for a predefined set of structural, functional, and valvular abnormalities (Supplemental Table 3). Sensitivity was highest for mitral regurgitation (80.9%) and tricuspid regurgitation (88.6%), consistent with their high prevalence in the study cohort, whereas specificity was generally high for LV dilation (94.5%), RV dilation (92.9%), and aortic stenosis (98.0%). Agreement measured by Cohen's kappa was highest for LV wall motion (κ = 0.438) and LV dilation (κ = 0.418). These findings suggest that structural and wall motion abnormalities were more consistently preserved in retrieved reports than valvular abnormalities, providing a direct assessment of concordance between retrieved and ground-truth reports.

We further evaluated the relationship between finding prevalence and retrieval performance across findings (Supplemental Figure 3). R@10 was inversely correlated with finding prevalence (Spearman *r* = −0.68, *P* = 0.008). These results suggest that retrieval performance was not primarily driven by finding prevalence in the test cohort.

### Effect of pretraining

Beyond input modality and multiview capability, the choice of pretrained models is a critical factor. For models with either image or video inputs, an important challenge is the imbalance in available pretrained resources: Image models benefit from extremely large-scale pretraining datasets, whereas video models have historically had access to much smaller ones. To evaluate this effect, we trained SingleImage and MultiVideo-4CH models using multiple pretrained weights—ImageNet-21k, LAION-2B, Kinetics-400, and VideoMAE2—and compared retrieval performance. VideoMAE2 differs from ViViT in architecture and uses 16 frames per video. This analysis was designed to isolate the effect of pretrained representations, and therefore used a consistent dataset split across all pretraining conditions.

As shown in Figure 4, pretraining substantially improved performance for both image- and video-based models. Large-scale image pretraining greatly narrowed the performance gap between image and video models that was observed when both were trained from scratch. However, the best performance was achieved by the video model pretrained with VideoMAE2, surpassing even the EchoCLIP-pretrained image model.

**Figure 4:**
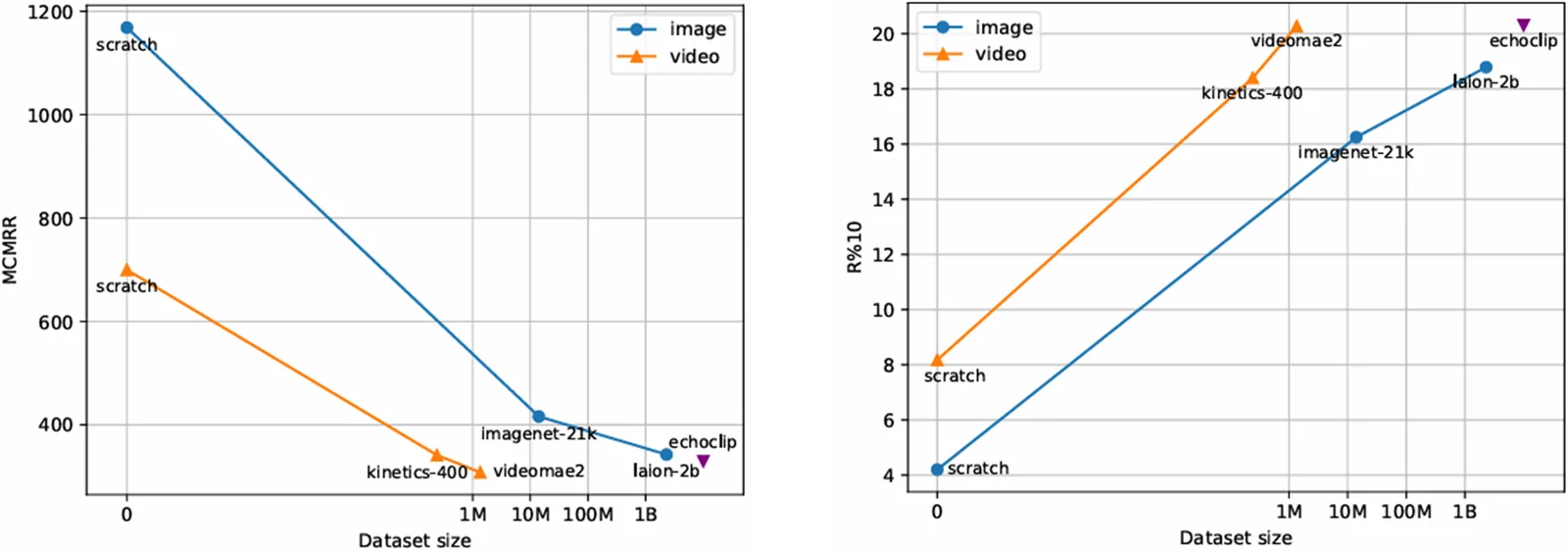
Effect of Pretrained Initialization on Cross-Modal Retrieval Performance Retrieval performance of image-based (SingleImage) and video-based (MultiVideo-4CH) models trained with different pretrained initializations is shown as a function of pretraining dataset size (log scale). Performance is evaluated using mean cross-modal retrieval rank (MCMRR) and recall at top 10 (R@10). Video-based models pretrained with VideoMAE2 achieved the best performance, highlighting the importance of domain-appropriate video pretraining. Models trained from scratch are shown for reference. 4CH = 4-chamber view.

Performance scaled almost linearly with video dataset size (log scale), suggesting that pretraining on 10 million to 100 million video samples could yield further gains. In contrast, image models exhibited a performance plateau, with only modest improvements observed even when trained on datasets containing several billion images.

### External evaluation using the EchoNet-Dynamic dataset

Given that the VideoMAE2-pretrained video model demonstrated the best retrieval performance among all evaluated models (Figure 4), we further assessed model robustness using an independent public dataset. Reference categories for LV dilation were derived from EDV, whereas LV wall motion categories were derived from EF, and these classifications were compared with report-derived classifications (Table 4).

**Table 4:** Agreement Between Generated Reports and Quantitative Echocardiographic Measurements in EchoNet-Dynamic

Model	LV Dilation, Accuracy (%)	LV Dilation, Weighted κ	LV Wall Motion, Accuracy (%)	LV Wall Motion, Weighted κ
VideoMAE2-pretrained video model	80.0 (72.0-88.0)	0.467 (0.108-0.691)	60.0 (51.0-70.0)	0.478 (0.287-0.650)
SingleVideo	58.0 (48.0-67.0)	0.289 (0.013-0.492)	55.0 (45.0-65.0)	0.320 (0.108-0.515)
SingleImage	62.6 (53.5-71.7)	0.207 (−0.006-0.403)	48.0 (38.0-58.0)	0.121 (−0.067-0.303)

Values represent agreement between report-derived severity categories and quantitative echocardiographic measurements in 100 cases from the EchoNet-Dynamic dataset. Left ventricular (LV) dilation categories were defined using end-diastolic volume and end-systolic volume, whereas LV wall motion categories were defined using ejection fraction. Accuracy is presented as percentages, and weighted kappa (κ) was used to assess agreement beyond chance. Values in parentheses indicate 95% CIs estimated using nonparametric bootstrap resampling (2,000 iterations).

For LV dilation assessment, the VideoMAE2-pretrained video model achieved the highest agreement with the reference categories, with an accuracy of 80.0% (95% CI: 72.0%-88.0%) and a weighted κ of 0.467 (95% CI: 0.108-0.691). In comparison, the SingleVideo and SingleImage models achieved accuracies of 58.0% and 62.6%, respectively, with lower weighted κ values.

Similarly, for LV wall motion assessment, the VideoMAE2-pretrained video model demonstrated the highest agreement, achieving an accuracy of 60.0% (95% CI: 51.0%-70.0%) and a weighted κ of 0.478 (95% CI: 0.287-0.650). These findings suggest that the top-performing model generates clinically coherent report outputs that remain consistent with quantitative echocardiographic measurements in an independent public dataset.

### Expert evaluation of generated reports

Three board-certified cardiologists independently evaluated reports generated by the VideoMAE2-pretrained video model (Supplemental Table 4). The generated reports received favorable evaluations across all domains. Median scores were 4 (IQR: 3-4) for diagnostic accuracy, completeness, and clinical usefulness. Mean scores ranged from 3.48 to 3.60, indicating that the generated reports were considered clinically acceptable and potentially useful for supporting echocardiographic interpretation.

## Discussion

The most prominent finding of this study was the approximately 2-fold improvement in performance achieved by using video rather than still images as input. Prior still image–based echocardiography AI systems have inherent limitations, as they do not adequately capture temporal dynamics that reflect the cardiac cycle.^22^ In clinical practice, the core of echocardiographic diagnosis relies on temporal information such as leaflet motion, regional wall motion, changes across the cardiac cycle, and subtle rhythm-dependent variations—elements consistently emphasized in multiple guidelines and prior studies.^1^^,^^2^^,^^23^^,^^24^ The superior performance of video-based models in our study quantitatively supports this long-standing clinical understanding. As shown in the Central Illustration, integration of temporal dynamics and multiple echocardiographic views enhances cross-modal retrieval performance compared with conventional approaches.

**Central Illustration:**
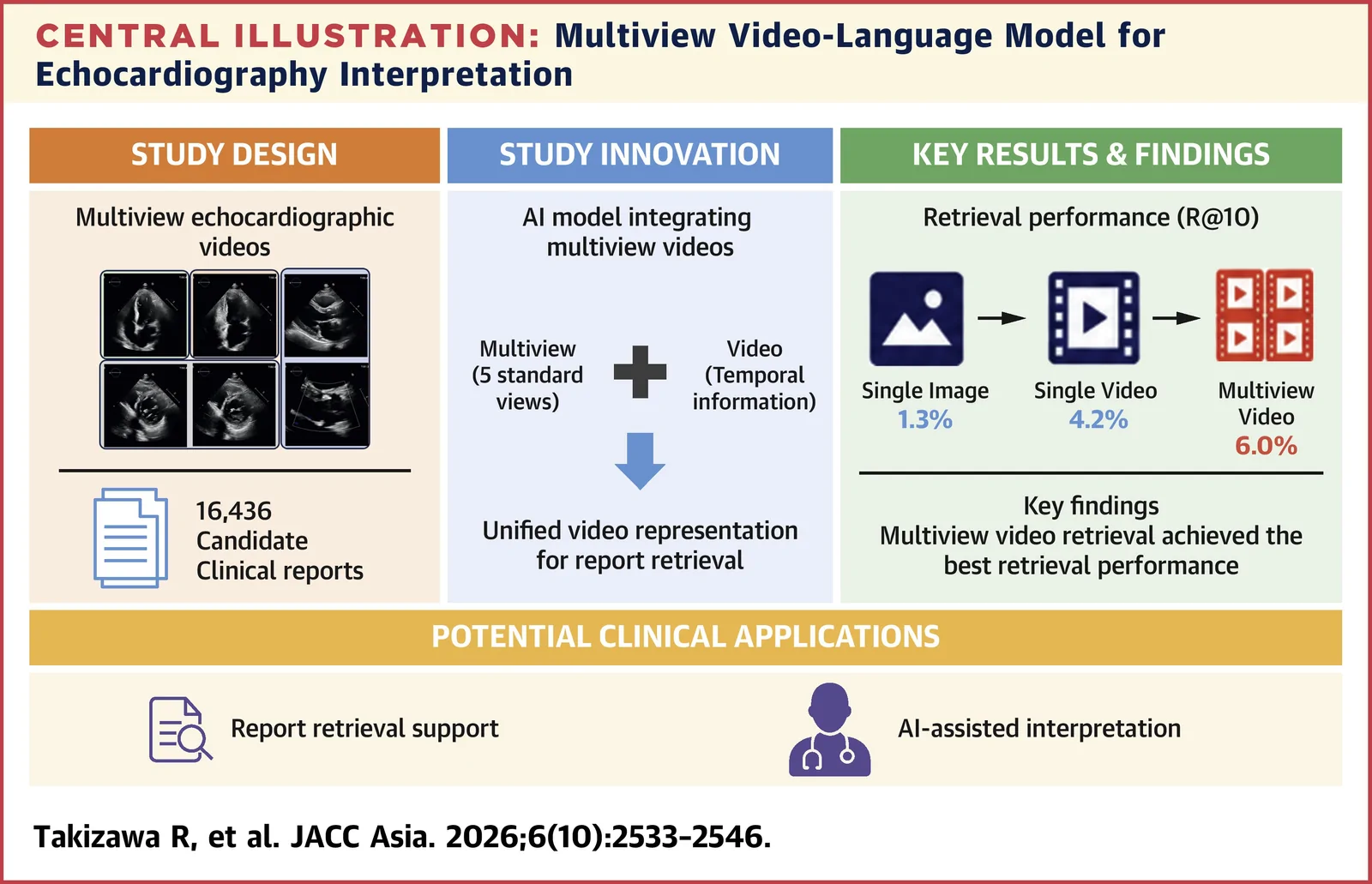
Multiview Video-Language Model for Echocardiography Interpretation This illustration summarizes a multiview video-language framework for automated echocardiography interpretation. Multiple video clips from each examination are encoded into a shared representation and aligned with clinical reports using contrastive learning. During inference, the most relevant report is retrieved based on similarity. Integration of multiview and temporal information improves retrieval performance compared with conventional approaches. AI = artificial intelligence.

Furthermore, the approximately 1.2-fold improvement observed with multiview input closely aligns with the practical realities of echocardiographic evaluation. Standard views—including the LAX, SAX, 2CH, 3CH, and 4CH views—each provide unique and complementary anatomical and functional information. Guidelines clearly state that final diagnostic conclusions should be based on a synthesis of these diverse views.^1^^,^^2^^,^^25^ The present findings demonstrate that an AI model can appropriately learn and leverage this multiperspective approach, suggesting that multiview integration captures clinically relevant information that may be useful for future decision-support applications.^3^^,^^4^ In addition, our analysis of performance according to the number of available views (Supplemental Figure 1) provides further insight into the role of multiview integration. While performance generally improved as the number of views increased, variability across different view combinations was observed, indicating that some views may contribute more strongly than others for specific findings. Because embeddings from different views were combined using simple averaging, future attention-based aggregation strategies may further improve performance. Analysis stratified by clinical findings (Supplemental Figure 2) demonstrated that the degree of performance improvement with increasing view numbers varied across findings. In particular, findings primarily characterized by morphological or motion-related features, such as LV wall motion abnormalities or dilation, showed more consistent improvement, whereas others, such as aortic stenosis, exhibited lower overall performance and less pronounced gains. This likely reflects differences in the extent to which each finding can be inferred from standard echocardiographic video inputs alone.

A concurrent study, EchoPrime, has similarly extended a CLIP-based framework to incorporate multiview and video inputs.^9^ However, the relative contributions of video information, multiview integration, and pretrained model selection were not systematically examined. In contrast, our study explicitly disentangled these components by constructing 3 ablation models, enabling controlled comparison of the effects of video input, multiview aggregation, and pretrained representations. This analytic approach clarifies which elements most strongly drive performance gains and provides practical insight for the development of future video-language echocardiography models.

In addition to dissecting the contributions of video information and multiview aggregation, our findings highlight the importance of pretrained representations. Image models benefited from exceptionally large pretrained datasets such as ImageNet-21k and LAION-2B, which substantially narrowed the performance gap relative to video models when all models were trained from scratch. However, the best retrieval performance was ultimately achieved by the video model initialized with VideoMAE2-pretrained weights, demonstrating that domain-appropriate video pretraining can surpass even very strong image-based initializations. These results suggest that future advances in video-language echocardiography models may depend on large-scale video pretraining corpora.

Another key aspect of this work is the use of over 700,000 echocardiographic videos paired with clinical reports for training. AI models trained on large-scale datasets encompassing the natural variability of real-world practice—including fluctuations in image quality, probe angulation, and patient characteristics—have the potential to reduce interpretive variability and provide more reproducible support. Such representations may serve as a foundation for clinical decision-support systems, although substantial improvements in retrieval performance and prospective validation will be required before routine clinical deployment.^26^

Taken together, this study demonstrates that incorporating both temporal dynamics and multiview information—2 foundational elements of echocardiographic diagnosis—substantially enhances interpretation performance compared with conventional approaches. Finding-level diagnostic analyses further showed moderate agreement for LV dilation and wall motion abnormalities. High specificity was observed for LV dilation, LV wall motion abnormalities, RV dilation, asynergy, and aortic stenosis, whereas mitral and tricuspid regurgitation showed high sensitivity. These findings suggest that retrieved reports reflected clinically meaningful echocardiographic information. As with any clinical decision-support system, such models should be viewed as complementary to—not replacements for—expert interpretation. Although the present results demonstrate methodological advances in multimodal representation learning, further improvements in retrieval performance and prospective clinical validation will be required before routine clinical deployment. Integration into real-world workflows will require evaluation of safety, user interaction, and interpretability.

### Study limitations

First, the model was trained on data derived from a single health care system. Although we performed a patient-level split cohort analysis and additionally evaluated the model using 100 cases from the publicly available EchoNet-Dynamic dataset, the possibility of institutional, workflow-specific, and language-related bias cannot be excluded. The EchoNet evaluation was limited to apical 4-chamber videos and quantitative measurements and did not constitute full external validation using independently curated echocardiographic reports. In addition, temporal holdout validation was not performed, and exclusion of videos with low view-classification confidence may have introduced selection bias toward higher-quality examinations. Therefore, further validation across multiple institutions, imaging protocols, equipment vendors, reporting styles, and languages will be necessary to establish generalizability. Second, the ground-truth labels used for contrastive learning were obtained from clinical reports. These reports inevitably contain interobserver variability, occasional documentation inconsistencies, and potential variability in reporting styles, which may introduce label noise into model training. Third, although our dataset included multiple standard echocardiographic views, certain rare pathologies and infrequently acquired views were under-represented. Future work should incorporate dedicated data collection for uncommon conditions and specialized imaging scenarios. Finally, model performance was based on a single training run, without seed-sensitivity analysis.

## Conclusions

This study demonstrates that incorporating video inputs and multiview information substantially improves vision-language–based echocardiographic interpretation. By extending a CLIP-based framework beyond conventional single-image, single-view settings, we show that retrieval performance improves when temporal dynamics and complementary views are jointly leveraged. These findings highlight the importance of video-language models that extract clinically relevant information from limited echocardiographic data, a capability that will be increasingly critical given constraints on medical data sharing. Future multi-institutional and prospective validation will be important to establish its generalizability and clinical utility.

### Code Availability

The source code used for model training and evaluation is publicly available.^27^

## Funding Support and Author Disclosures

This work was supported by the Cross-ministerial Strategic Innovation Promotion Program (SIP) on ‘Integrated Health Care System’ (grant number: JPJ012425) and by a Japanese Circulation Society Research Grant for Artificial Intelligence in Cardiovascular Medicine (2024). The authors have reported that they have no relationships relevant to the contents of this paper to disclose.

## Appendix

Supplemental Equations, Figures, and Tables
