# Supplementary material for: Comprehensive Echocardiography Interpretation Using Video and Multiview Vision-Language AI
Source: JACC Asia. 2026 Aug 27;6(10):2533–46. doi: 10.1016/j.jacasi.2026.06.039 (PMC13700026; doi:10.1016/j.jacasi.2026.06.039)
Supplement: Supplementary file 1 [file mmc1.docx]

**Supplemental Material**

Supplemental Equation S1 (Video-to-Report Term)

Let $v_{i}$ and $t_{i}$ denote the L2-normalized video and text embeddings for the i-th matched pair in a mini-batch of size B. Cosine similarity is used to measure the alignment between embeddings, and a temperature parameter τ is introduced to scale the similarity scores in the contrastive loss. The video-to-report contrastive loss is defined as follows:

$$\begin{aligned} \begin{aligned} \mathcal{L}_{\text{video-to-report}}=\frac{1}{B}\sum_{i=0}^{B} -\log\frac{\exp\left( \frac{1}{\tau}\frac{t_{i}^{T}v_{i}}{|t_{i}||v_{i}|} \right)}{\sum_{i} \exp\left( \frac{1}{\tau}\frac{t_{i}^{T}v_{i}}{|t_{i}||v_{i}|} \right)} \end{aligned}\left( 1 \right) \end{aligned}$$

Supplemental Equation S2 (Report-to-Video Term)

Conversely, the report-to-video loss is defined as:

$$\begin{aligned} \begin{aligned} \begin{aligned} \mathcal{L}_{report-to-video}=\frac{1}{B}\sum_{i=0}^{B} -\log\frac{\exp\left( \frac{1}{\tau}\frac{v_{i}^{T}t_{i}}{|v_{i}||t_{i}|} \right)}{\sum_{i} \exp\left( \frac{1}{\tau}\frac{v_{i}^{T}t_{i}}{|v_{i}||t_{i}|} \right)} \end{aligned} \end{aligned}\#\left( 2 \right) \end{aligned}$$

The overall contrastive loss used for training is the mean of Eq. 1 and Eq. 2.

Supplemental Equation S3

The entire report retrieval process based on multi-view video interpretation is summarized in Algorithm 1.

Supplemental Figure 1. Model performance according to the number of available views.

Each dot represents a unique combination of echocardiographic views, and the solid line indicates the mean performance at each level. Retrieval performance (R@10) generally increased with the number of available views. However, variability within each group indicates that performance depends not only on the number of views but also on the specific combination of views.

Supplemental Figure 2. Retrieval performance by number of available views across selected clinical findings.

Mean retrieval performance (R@10) is shown according to the number of available echocardiographic views for selected clinical findings. Each point represents a unique combination of views, and solid lines indicate the mean performance at each view count. Performance generally improved with increasing numbers of available views, although variability across view combinations was observed. The magnitude and consistency of performance gains differed across findings.

Supplemental Figure 3. Relationship between finding prevalence and retrieval performance

R@10 values for video-to-report retrieval among examinations positive for each echocardiographic finding were plotted against the prevalence of that finding in the test cohort. Each point represents a predefined echocardiographic finding, and error bars indicate 95% confidence intervals estimated using patient-level bootstrap resampling with 2,000 iterations. The dashed line represents the fitted linear regression line. Retrieval performance was inversely correlated with finding prevalence (Spearman ρ = −0.68, P = 0.008), indicating that retrieval performance did not systematically decrease for less prevalent findings.

Supplemental Table 1. Performance of the echocardiographic view-classification CNN

| View | AUC | PPV (%) | Sensitivity (%) |
| --- | --- | --- | --- |
| LAX | 0.994 | 99 | 98 |
| SAX | 0.998 | 98 | 96 |
| 2CH | 0.999 | 97 | 100 |
| 3CH | 1.000 | 100 | 97 |
| 4CH | 1.000 | 99 | 100 |
| Other | 0.996 | 93 | 95 |

**Overall accuracy: 97.8%**

Performance of the internally developed convolutional neural network used for echocardiographic view classification during preprocessing. The classifier assigned videos to one of five standard echocardiographic views (LAX, SAX, 2CH, 3CH, and 4CH) or to the “Other” category. Performance was evaluated in an independent validation dataset. AUC indicates area under the receiver-operating characteristic curve, PPV positive predictive value. The overall classification accuracy was 97.8%.

Supplemental Table 2. Pairwise bootstrap comparisons of retrieval performance metrics

Part A. Video-to-report retrieval (V→R)

| Comparision | ΔMCMRR | ΔR@1 (%) | ΔR@5 (%) | ΔR@10 (%) |
| --- | --- | --- | --- | --- |
| MultiVideo vs MultiVideo-4CH | -303 (-325 to -281) | +0.2 (0.0-0.3) | +0.8 (0.6-1.1) | +1.4 (1.1-1.7) |
| MultiVideo vs SingleVideo | -334 (-373 to -298) | +0.1 (-0.1-0.3) | +1.0 (0.7-1.3) | +1.8 (1.4-2.2) |
| MultiVideo vs SingleImage | -2184 (-2248 to -2120) | +0.7 (0.5-0.8) | +2.8 (2.5-3.1) | +4.7 (4.3-5.1) |

Part B. Report-to-Video retrieval (R→V)

| Comparision | ΔMCMRR | ΔR@1 (%) | ΔR@5 (%) | ΔR@10 (%) |
| --- | --- | --- | --- | --- |
| MultiVideo vs MultiVideo-4CH | -299 (-319 to -280) | +0.4 (0.3-0.6) | +1.4 (1.1-1.7) | +2.0 (1.7-2.3) |
| MultiVideo vs SingleVideo | -425 (-461 to -393) | +0.5 (0.3-0.7) | +1.8 (1.5-2.2) | +2.8 (2.4-3.2) |
| MultiVideo vs SingleImage | -1936 (-1992 to -1880) | +0.8 (0.6-1.0) | +2.8 (2.4-3.1) | +4.4 (4.0-4.8) |

Pairwise bootstrap comparisons of retrieval performance metrics between models. Positive values for R@K indicate higher retrieval performance of MultiVideo relative to the comparison model and are reported as absolute percentage-point differences. Negative values for mean cross-modal retrieval rank (MCMRR) indicate superior retrieval performance because lower MCMRR values represent better retrieval. Values are presented as point estimates with 95% confidence intervals (CIs), estimated using non-parametric patient-level bootstrap resampling with 2,000 iterations. V→R indicates video-to-report retrieval, and R→V indicates report-to-video retrieval.

Supplemental Table 3. Agreement Between Echocardiographic Findings Extracted from Retrieved Reports and Ground-Truth Reports

| Finding | Sensitivity (%) | Specificity (%) | PPV (%) | NPV (%) | Cohen’s κ |
| --- | --- | --- | --- | --- | --- |
| LV dilation | 45.6 (42.6-48.6) | 94.5 (94.0-94.9) | 50.4 (47.2-53.7) | 93.4 (92.8-93.9) | 0.418 (0.390-0.447) |
| LV WM | 52.7 (50.2-55.1) | 91.3 (90.7-91.8) | 52.2 (49.4-54.7) | 91.4 (90.7-92.1) | 0.438 (0.413-0.460) |
| Asynergy | 25.5 (23.2-27.8) | 94.4 (94.0-94.8) | 32.8 (29.7-36.0) | 92.2 (91.6-92.8) | 0.221 (0.196-0.246) |
| AS | 10.8 (8.3-13.3) | 98.0 (97.8-98.2) | 16.5 (12.6-20.5) | 96.8 (96.3-97.1) | 0.106 (0.076-0.135) |
| AR | 52.2 (51.2-53.8) | 67.2 (66.2-68.2) | 54.1 (52.6-55.6) | 65.7 (64.5-67.0) | 0.198 (0.181-0.214) |
| MR | 80.9 (80.2-81.6) | 44.1 (42.1-46.0) | 88.5 (87.9-89.1) | 30.2 (29.6-31.7) | 0.210 (0.193-0.228) |
| TR | 88.6 (88.0-89.1) | 30.1 (27.6-32.7) | 93.6 (93.1-94.0) | 18.7 (16.9-20.4) | 0.146 (0.125-0.166) |
| RV dilation | 40.0 (36.0-43.9) | 92.9 (92.4-93.3) | 27.2 (23.8-30.5) | 95.9 (95.4-96.3) | 0.269 (0.234-0.303) |

Sensitivity, specificity, positive predictive value (PPV), negative predictive value (NPV), and Cohen's kappa were calculated by comparing findings extracted from the retrieved reports with those in the corresponding ground-truth reports. Values are presented with 95% confidence intervals estimated using patient-level bootstrap resampling. LV dilation indicates left ventricular dilation; LV WM, any left ventricular wall motion abnormality; Asynergy, regional wall motion abnormality; RV dilation, right ventricular dilation; AS, aortic stenosis; AR, aortic regurgitation; MR, mitral regurgitation; and TR, tricuspid regurgitation.

Supplemental Table 4. Expert evaluation of reports generated by the VideoMAE2-pretrained video model from EchoNet-Dynamic cases.

| Metric | Mean ± SD | Median (IQR) |
| --- | --- | --- |
| Diagnostic accuracy | 3.48 ± 0.94 | 4 (3-4) |
| Completeness | 3.60 ± 0.83 | 4 (3-4) |
| Clinical usefulness | 3.49 ± 1.01 | 4 (3-4) |

Three board-certified cardiologists independently evaluated reports generated by the VideoMAE2-pretrained video model from 100 EchoNet-Dynamic cases (300 total evaluations). Each case consisted of an apical 4-chamber echocardiographic video with corresponding quantitative measurements. Reports were assessed using a 5-point Likert scale for diagnostic accuracy, completeness, and clinical usefulness. Scores ranged from 1 to 5, where 1 indicated poor performance, 2 fair performance, 3 acceptable performance, 4 good performance, and 5 excellent performance. Data are presented as mean ± standard deviation (SD) and median (interquartile range [IQR]).
